# Blind normalization of public high-throughput databases

**DOI:** 10.7717/peerj-cs.231

**Published:** 2019-11-11

**Authors:** Sebastian Ohse, Melanie Boerries, Hauke Busch

**Affiliations:** 1 Institute of Molecular Medicine and Cell Research, University of Freiburg, Freiburg, Germany; 2 German Cancer Consortium (DKTK), German Cancer Research Center (DKFZ), Heidelberg, Germany; 3 Institute of Medical Bioinformatics and Systems Medicine, Medical Center - University of Freiburg, Faculty of Medicine, University of Freiburg, Freiburg, Germany; 4 Institute of Experimental Dermatology, University of Lübeck, Lübeck, Germany

**Keywords:** Blind normalization, High-throughput data, Compressed sensing, Confounding factors

## Abstract

The rise of high-throughput technologies in the domain of molecular and cell biology, as well as medicine, has generated an unprecedented amount of quantitative high-dimensional data. Public databases at present make a wealth of this data available, but appropriate normalization is critical for meaningful analyses integrating different experiments and technologies. Without such normalization, meta-analyses can be difficult to perform and the potential to address shortcomings in experimental designs, such as inadequate replicates or controls with public data, is limited. Because of a lack of quantitative standards and insufficient annotation, large scale normalization across entire databases is currently limited to approaches that demand ad hoc assumptions about noise sources and the biological signal. By leveraging detectable redundancies in public databases, such as related samples and features, we show that blind normalization without constraints on noise sources and the biological signal is possible. The inherent recovery of confounding factors is formulated in the theoretical framework of compressed sensing and employs efficient optimization on manifolds. As public databases increase in size and offer more detectable redundancies, the proposed approach is able to scale to more complex confounding factors. In addition, the approach accounts for missing values and can incorporate spike-in controls. Our work presents a systematic approach to the blind normalization of public high-throughput databases.

## Introduction

**Figure 1:**
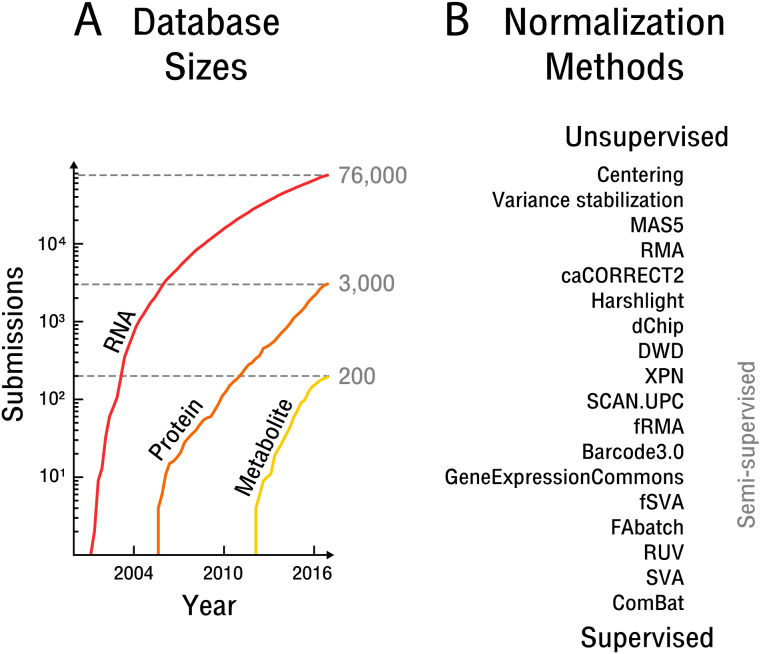
The rise of high-throughput technologies and associated normalization methods. (A) Submissions of RNA are based on NCBI’s Gene Expression Omnibus (Barrett et al., 2013), protein on EBI’s PRIDE database (Vizcaíno et al., 2016) and metabolite on EBI’s MetaboLights database (Haug et al., 2012). Notably, actual samples available are approximately an order of magnitude larger than the number of submissions. (B) Overview of common normalization methods from unsupervised to supervised learning.

In the current age of biological science an unprecedented amount of quantitative high-dimensional data has been acquired and needs to be analyzed. In particular, high-throughput technologies in the domain of molecular and cell biology, as well as medicine, have led to a rise in the quantification of biological molecules that underlie fundamental cellular processes, such as gene expression, metabolic flux and protein signaling (see Fig. 1A). These fundamental processes as a whole orchestrate and underpin the dynamics of the cell (Joyce & Palsson, 2006). Most of the acquired high-throughput data and particularly transcriptome data is submitted to public databases for re-analysis and reuse in research. Hence, researchers increasingly rely on samples from public databases to address shortcomings in experimental design, such as insufficient randomization or missing replicates. In addition, high-throughput data based meta-analyses are best performed with a large number of samples, such as across entire databases and different measurement technologies, in order to obtain insights applicable beyond a specific experimental setting. Thus, the development of data integration techniques is increasingly important. However, significant challenges remain.

The overarching problem for data integration is that of normalization, which is becoming more apparent and limiting as the need for reuse and re-analysis of high-throughput data increases. Normalization involves the attenuation of bias resulting from confounding factors affecting the measurement process. Technical bias of an instrument or sample preparation procedure can be addressed by measuring identically processed quantitative standards. Use of such standards is widespread in serial technologies. The further up-stream in the measurement process quantitative standards are introduced, the more potential sources of bias can be accounted for. Biological bias due to non-identical cells or organisms is often addressed instead by randomization (Montgomery, 2008). This later approach presupposes that the contrast of interest and potential bias sources are known. An overview of potential bias sources with a focus on high-throughput technologies is given by Lazar et al. (2012). High-throughput technologies are challenging to normalize especially because the bias of biological molecules measured in parallel is not independent. Such non-independent bias stems from molecular interactions throughout the measurement process, including sample preparation procedures and instrument settings that are dependent on the measured sample itself and its biological signal. Quantitative measurement standards must therefore effectively cover a vast number of possible combinations of potential signal measured. In addition, measurement process or instrument components are sometimes one-time-use, such as in the case of microarray technologies, making appropriate normalization with measurement standards unfeasible. In part for these reasons, high-throughput technologies have been designed with a focus on relative comparisons, such as fold changes, rather than absolute quantification. While a limited number of spike-in standards can account for some technical bias (Lovén et al., 2012) sample preparation procedures that are important sources of bias, such as library preparation, protein extraction or metabolic labeling, generally happen up-stream of spike-in addition. Bias attenuation by randomization is not generally possible, as contrasts of interest are not initially known in the exploratory analyses typically performed with high-throughput technologies.

The initial experimental design establishes how quantitative measurement standards or randomization are employed in a particular experiment. However, in the case of experiments that draw on samples from public databases, the attenuation of bias must be done *post hoc*. Attempts at such normalization have produced different methods across the spectrum of unsupervised to supervised learning (see Fig. 1B).

Unsupervised approaches generally make use of ad hoc assumptions about noise sources or the biological signal, which are then leveraged in an attempt to average out bias. While early methods were concerned with simple centering and scaling (Cheadle et al., 2003), more recent approaches assume that an appropriate scaling is obtained by scaling across features, such as through variance stabilization (Huber et al., 2002), or by scaling across samples, such as through quantile normalization (Bolstad et al., 2003; Irizarry et al., 2003). The later approach is widely used but requires the assumption that the overall biological signal does not vary significantly between samples. Another major drawback is that unsupervised approaches fail to exploit the wealth of information available in public high-throughput databases.

Semi-supervised approaches implicitly or explicitly exploit additional data to learn parameters that can then be transferred to the dataset at hand. In particular, frozen SVA (Parker, Corrada Bravo & Leek, 2014), frozen RMA (McCall et al., 2011) and the Gene Expression Commons (Seita et al., 2012) take such an approach. The later methods aim to adjust the weight and scale parameters of the measured features based on global distributions obtained by the use of additional data. However, the frozen SVA method requires prior knowledge of the contrast of interest for the additional data to be of use and is therefore impractical in the case of exploratory analyses. The frozen RMA approach is based on quantile normalization and thus makes equally restrictive assumptions about the biological signal.

Supervised approaches make use of replicate samples or prior knowledge of potential confounding factors and contrasts of interest. If the contrast of interest has replicate samples overlapping with known confounding factors, these replicates can subsequently be used to remove bias; for example, through simple centering (Li & Wong, 2001) or more complex non-linear adjustments (Benito et al., 2004). In the case of small sample sizes, the popular empirical Bayes method ComBat (Johnson, Li & Rabinovic, 2007) can be applied. However, any supervised methods is unable to detect and remove bias outside of a setting that includes replicate samples specifically designed to limit known confounding factors, or alternatively, prior knowledge of the contrast of interest. Unfortunately, as annotation of high-throughput data with respect to sample information and the experimental protocol used is often insufficient and too incoherent for machine processing, supervised approaches to normalization are generally unfeasible for public databases.

The blind compressive normalization algorithm developed here makes use of the sparsity assumption combined with the identification and use of detectable redundancies in high-throughput databases to normalize for unknown confounding factors. The sparsity assumption is the well motivated assumption that signals of interest generally lie on low dimensional manifolds (Hastie, Tibshirani & Wainwright, 2015). In the framework of compressed sensing it enables blind recovery of bias and subsequent normalization of high-throughput databases from merely estimated redundancies, such as correlations in that data. Compressed sensing is a recent field that studies the ability to accurately reconstruct signals of interested based on very few measurements (below the Nyquist sampling rate) (Candès & Wakin, 2008). We sidestep more restrictive assumptions on the biological signal or noise sources common in unsupervised normalization approaches and do not require prior knowledge of the contrast of interest or appropriate sample annotation as required for supervised normalization approaches.

For the biological or medical researcher working with high-throughput data this means that when blind compressive normalization can be successfully applied to a database that includes their samples of interest, these samples are subsequently more comparable to each other and overall to other samples in the database, as bias stemming from unknown confounding factors is attenuated.

## Methods

The challenge of normalizing large high-throughput databases is distinct from the traditional p ≫ n problem (Friedman, Hastie & Tibshirani, 2001) often encountered in high-throughput data normalization. The number of features (p) and the number of samples (n) in public high-throughput databases is currently large and on the same order of magnitude (p ≈ n). Therefore, computational scalability becomes an important consideration. Recent advances in the field of machine learning, based on the sparsity assumption, have shown that limited sampling of high-dimensional data is often sufficient for efficient signal recovery. For example, in the area of collaborative filtering, large low-rank matrices are routinely recovered from a small number of sampled entries (Mazumder, Hastie & Tibshirani, 2010; Jain, Netrapalli & Sanghavi, 2013; Vandereycken, 2013). If confounding factors in high-throughput databases are equally amenable to the sparsity assumption, bias due to the measurement process may be recoverable from a relatively small number of measured quantitative standards. Since such standards are not available or feasible to obtain *post hoc*, we propose instead to utilize database wide redundancies to obtain the necessary constraints that enable bias recovery and subsequent normalization.

**Figure 2:**
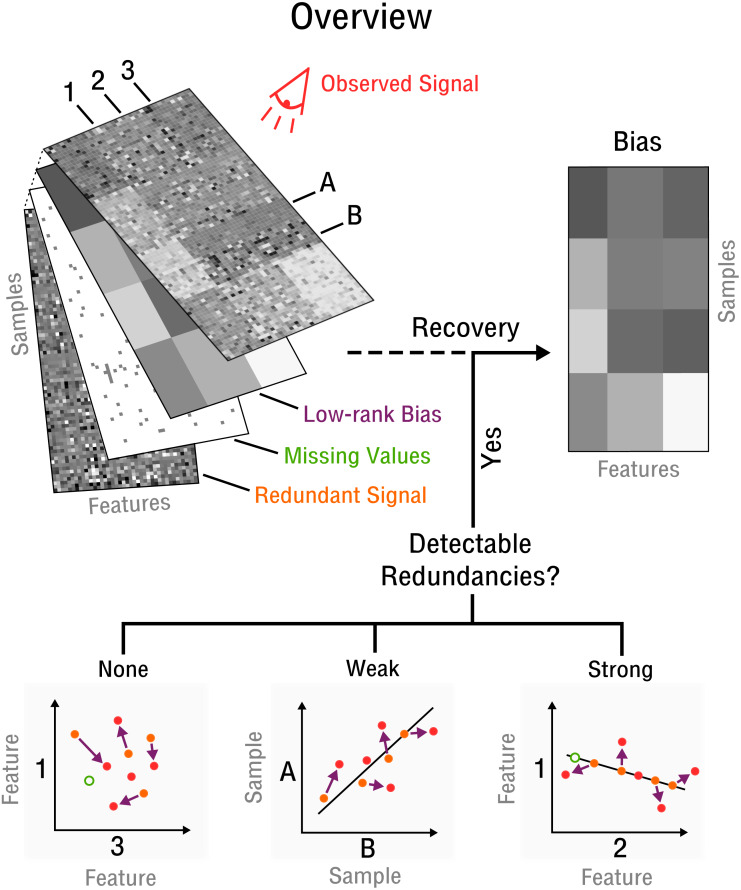
Blind recovery of bias. A database consisting of features, such as measurements of RNA, protein or metabolite and samples, such as different cell types under various stimuli, is observed. Recovery of the underlying bias (purple) is feasible if some redundant underlying signal (orange) exists that is incoherent to the bias and partially detectable by observation (red). Redundancies can be categorized as detectable and as weak or strong based on the correlation strength between features or samples. The more redundant a signal is the closer it falls on the perfect correlation line.

Our approach begins with the assumption that there are a limited number of confounding factors that markedly affect the measurement process. Thus, the bias is modeled as a low-dimensional manifold that takes the form of low-rank matrix (see Fig. 2) denoted as **X**. This is a flexible model which can approximate arbitrarily close any underlying bias. Opposed to traditional signal recovery approaches, we specifically model the bias (systematic noise) instead of the potentially complex signal. In the framework of compressed sensing the respective matrix recovery problem resulting in the recovery of **X**, is defined as follows (Tan et al., 2014).

Definition 1 Given a linear operator 𝒜:ℝ^*n*×*m*^ → ℝ^*p*^, let $\mathbf{y}=\mathcal{A}(\mathbf{X})+\mathrm{&epsi;}$ be a set of *p* measurements of an unknown rank $\hat {r}$ matrix **X** ∈ ℝ^*n*×*m*^ and noise *ϵ*. Matrix recovery solves the problem of ${\min }_{\mathbf{X}}{ \left\| \mathbf{y}-\mathcal{A}(\mathbf{X}) \right\| }_{2}^{2}$ subject to ${rank} \left( \mathbf{X} \right) \leq r$, where *p* ≪ *nm* and $r\geq \hat {r}$.

The specific type of linear operator used depends on the context and is commonly defined as the Frobenius inner product of **X** and sensing matrices {**A**_*i*_ ∈ ℝ^*n*×*m*^}_*i*=1,…,*p*_ such that ${\mathbf{y}}_{i}={\mathop{\sum }\nolimits }_{j=1}^{n}{\mathop{\sum }\nolimits }_{k=1}^{m}({\mathbf{A}}_{i})_{jk}{\mathbf{X}}_{jk}$. In the general case of *dense* sensing, for which various recovery guarantees have been established (Candes & Plan, 2011), sensing matrices **A**_*i*_ are defined ∀*j* ∈ {1, …, *n*} and ∀*k* ∈ {1, …, *m*} as $({\mathbf{A}}_{i})_{jk}&sim; \mathcal{N}$. However, this approach at bias recovery presupposes a measurement setup that provides constraints (e.g., prior information) about **A**_*i*_ and **y**_*i*_ to recover **X** according to Definition 1. Such prior information is typically not available, but we show that it can be indirectly obtained from an approximation of the redundancies that commonly exists in high-throughput databases (see ‘Blind recovery’). But first, before focusing on the case of blind recovery, we introduce the intermediate case of k-sparse recovery of which blind recovery is an extension.

### K-sparse recovery

Several modifications to the traditional approach of matrix recovery through *dense* sensing exist, including row and column only or rank-1 based sensing matrices (Wagner & Zuk, 2015; Cai & Zhang, 2015; Zhong, Jain & Dhillon, 2015). The common case of *entry* sensing can be seen as a special case of *dense* sensing (Candes & Plan, 2010) that requires additional assumptions for guaranteed recovery and knowledge of specific entries of **X**. It is the simplest form of k-sparse recovery, where each sensing matrix is 1-sparse (contains only one nonzero entry). If sufficient quantitative standards or spike-ins were available to obtain estimates at specific nonzero entries Ω_(*s*_1_,*t*_1_)_ of **X** from high-throughput databases, then *post hoc* bias recovery through *entry* sensing would be possible, with *s*_1_ ∼ Uniform({1, …, *n*}), *t*_1_ ∼ Uniform({1, …, *m*}) and **y**_*i*_ = **X**_*s*_1_*t*_1__. In this case the 1-sparse sensing matrices **A**_*i*_ are defined as:

(1) \begin{eqnarray*}({\mathbf{A}}_{i})_{jk} \left\{ \begin{array}{@{}ll@{}} \displaystyle \sim 1 &\displaystyle \text{if}(j,k)=({s}_{1},{t}_{1})\\ \displaystyle =0 &\displaystyle \text{otherwise} \end{array} \right. \end{eqnarray*}

The next level of complexity of k-sparse recovery is a 2-sparse sensing matrix based approach, with entries Ω_(*s*_1_,*t*_1_)(*s*_2_,*t*_2_)_ chosen uniformly at random as before and (*s*_1_, *t*_1_) ≠ (*s*_2_, *t*_2_). In this case the 2-sparse sensing matrices **A**_*i*_ are defined as:

(2) \begin{eqnarray*}({\mathbf{A}}_{i})_{jk} \left\{ \begin{array}{@{}ll@{}} \displaystyle \sim \mathcal{N} &\displaystyle \text{if}(j,k)\in \{({s}_{1},{t}_{1}),({s}_{2},{t}_{2})\}\\ \displaystyle =0 &\displaystyle \text{otherwise} \end{array} \right. \end{eqnarray*}

Analogously as for the *dense* sensing approach, k-sparse recovery presupposes a measurement setup that provides prior information about **A**_*i*_ and **y**_*i*_ to recover **X**. It differs from *dense* sensing by the random sparsification of measurement operators (see Eq. (2)). We use k-sparse recovery as an intermediate step to blind recovery, where inaccuracies due to the additional estimation step of blind recovery are controlled for in order to allow simple evaluation (see ‘Results’).

### Blind recovery

**Figure 3:**
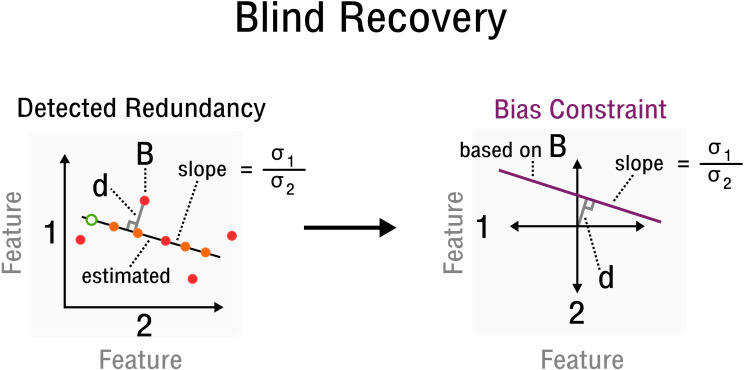
Measurement inference process from detected redundancies to bias constraints required for recovery. In feature space a redundancy is detected. A sample **B** allows the characterization of **d** and slope $ \frac{{\sigma }_{1}}{{\sigma }_{2}} $. The corresponding bias constraint based on **B** is denoted in this new feature space, where **d** characterizes the offset from the origin. All bias estimates are constrained by the given curve (purple).

In blind recovery we show how to estimate the necessary constraints (e.g., prior information) about **A**_*i*_ and **y**_*i*_ from the observed signal **O** (see Fig. 2). The 2-sparse sensing matrices **A**_*i*_ and respective measurements **y**_*i*_ are defined as:

(3) \begin{eqnarray*}({\mathbf{A}}_{i})_{jk} \left\{ \begin{array}{@{}ll@{}} \displaystyle =\hat {\sigma }({\mathbf{O}}_{{s}_{1}\ast }) &\displaystyle \text{if}(j,k)=({s}_{1},x)\\ \displaystyle =\hat {\sigma }({\mathbf{O}}_{{s}_{2}\ast }) &\displaystyle \text{if}(j,k)=({s}_{2},x)\\ \displaystyle =0 &\displaystyle \text{otherwise} \end{array} \right. \end{eqnarray*}

(4) \begin{eqnarray*}{\mathbf{y}}_{i}=\hat {\sigma }({\mathbf{O}}_{{s}_{2}\ast }){\mathbf{d}}_{{s}_{2}}-\hat {\sigma }({\mathbf{O}}_{{s}_{1}\ast }){\mathbf{d}}_{{s}_{1}}\end{eqnarray*}

where $\hat {\sigma }({\mathbf{O}}_{{s}_{1}\ast })$ and $\hat {\sigma }({\mathbf{O}}_{{s}_{2}\ast })$ are estimates of the standard deviation of the corresponding rows **O**_*s*_1_∗_ and **O**_*s*_2_∗_ of the observed signal, respectively. Specifically, the values for entries Ω_(*s*_1_,*x*)(*s*_2_,*x*)_ of 2-sparse sensing matrices **A**_*i*_ are determined by redundancy information, such as correlations between features and samples, which must be estimated from **O**. Furthermore, [**d**_*s*_1__, **d**_*s*_2__] is the orthogonal vector from point (**O**_*s*_1_*x*_, **O**_*s*_2_*x*_) to the line crossing the origin with slope $\hat {\sigma }({\mathbf{O}}_{{s}_{1}\ast })/\hat {\sigma }({\mathbf{O}}_{{s}_{2}\ast })$ in the space of rows **O**_*s*_1_∗_ and **O**_*s*_2_∗_ (see Fig. 3). Thus, **y**_*i*_ can be reconstructed from relative constraints encoded in the correlations of **O**. Without specifying an absolute value for a specific entry, but by specifying a correlation between two particular features, the bias is constrained by the line which goes through point (**O**_*s*_1_*x*_, **O**_*s*_2_*x*_), given that the observed matrix is centered. Since redundancies not only exist for features but also samples, the transpose of the observed signal **O**^*T*^ and its corresponding matrix entries ${\Omega }_{({s}_{A},v)({s}_{B},v)}^{T}$ are used equivalently. Thus, while *s*_1_∕*s*_*A*_ and *s*_2_∕*s*_*B*_ specify a correlated pair of rows/columns, *x*∕*v* specifies a particular observation in the space of that correlated pair (see Fig. 3). With linear operator **A**, bias **X** and measurements **y** defined accordingly, the standard matrix recovery problem given in Definition 1 is then solved by Riemannian optimization (Vandereycken, 2013), specifically with the Pymanopt implementation (Townsend, Koep & Weichwald, 2016).

### Simulation

We conduct a series of simulations to empirically evaluate the performance and robustness of the k-sparse recovery and blind recovery approaches. To this end a synthetic high-throughput database is generated (see Data Availability) by combining an underlying redundant signal **S** with a known low-rank bias **X** to be recovered. We generate the redundant signal **S** from a matrix normal distribution. This is a common model for high-throughput data (Allen & Tibshirani, 2012). Specifically, $\mathbf{S}&sim; {\mathcal{MN}}_{n&times; p}(\mathbf{M},{\mathbf{AA}}^{T},{\mathbf{B}}^{T}\mathbf{B})$, where **M** denotes the mean matrix and both **AA**^*T*^ and **B**^*T*^**B** denote the covariance matrices describing the redundancies in feature and sample space, respectively. Sampling is performed by drawing from a multivariate normal distribution $\mathbf{N}&sim; {\mathcal{MN}}_{n&times; p}(\mathbf{0},\mathbf{I},\mathbf{I})$ and transforming according to **S** = **M** + **ANB**. Importantly, different features and samples have different standard deviations, which are used in the construction of the covariance matrices (in combination with random binary block structured correlation matrices). Ideally, the standard deviations follow a sub-gaussian distribution (Candès & Wakin, 2008). Missing values are modeled according to missing at random (MAR) or missing not at random (MNAR) scenarios. The bias to be recovered is modeled as a random low-rank matrix **X** = *U*Σ*V*^*T*^ with Σ generated from diag(*σ*_1_, …, *σ*_*m*_). Eigenvalues are denoted as *σ* and are sampled from Uniform(0, 1). Matrix rank is denoted by *m*. Eigenvectors **U** and **V** are obtained from Stiefel manifolds generated by the QR decomposition of a random normal matrix (Townsend, Koep & Weichwald, 2016). Both redundant signal **S** and low-rank bias **X** are combined additively to yield the observed signal matrix **O** = **X** + **S**. The signal-to-noise ratio is kept approximately constant across bias matrices of different rank by scaling the eigenvalues of **X** to an appropriate noise amplitude.

**Figure 4:**
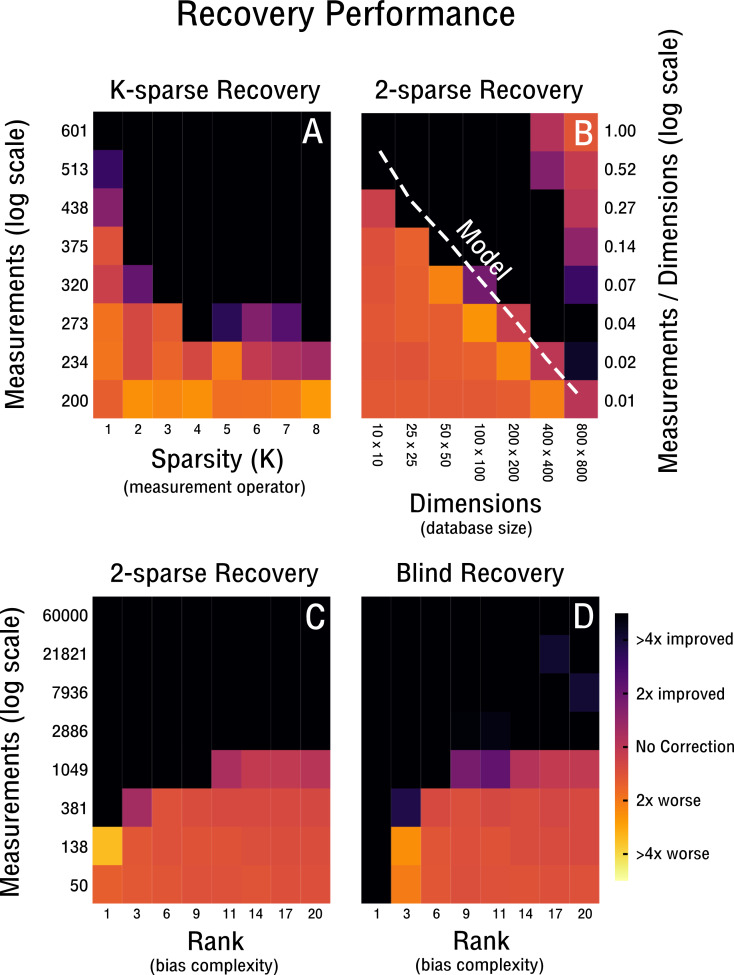
Performance of 2-sparse and blind recovery. (A) Decreasing the sparsity of the measurement operator from 2 to 10-sparse shows a leveling-off effect (rank-2, 50 × 50). (B) Scalability of 2-sparse recovery overlaid with model O(*c*_0_*r*(*n* + *m*)) (Wei et al., 2016) (white dashed line). The larger the high-throughput database the more likely is reconstruction of more complex noise structures from a small percentage of measurements (rank-2). (C, D) Evaluation of the proof-of-concept for the 2-sparse case and blind recovery of bias with increasing noise complexity (50 × 50).

## Results

### Recovery performance

Our performance evaluation starts with the case of k-sparse recovery shown in Figs. 4A–4C and derived in ‘K-sparse Recovery’. In our setup, the difference in measurement operator construction between sparse and *dense* sensing has little effect on the performance. Initial differences levels off rapidly as shown in Fig. 4A. Notably, in Fig. 4A we observe no significant difference in performance between a 4-sparse and 8-sparse measurement operator. The storage requirements for the *dense* sensing variant become prohibitive quickly (Cai & Zhang, 2015) and therefore we do not simulate above 8-sparse measurement operators. In Fig. 4B we highlight the advantageous scaling behavior of the 2-sparse approach. This allows reconstruction of bias from a small percentage of potential measurements of large high-throughput databases. Therefore, for databases on the order of tens of thousands of features and samples, only a small fraction of correlations need to be considered in order to reconstruct the low-dimensional model of the bias **X**. Thus, when estimating correlations and corresponding standard deviations from the data in the case of blind recovery, high-specificity and low-sensitivity estimators can be used; as high-sensitivity is not required with an overabundance of measurements and the focus can be placed on high-specificity instead. The non-perfect recovery in the top right of Fig. 4B is likely due to convergence failure of the conjugate gradient optimizer, because of a heavily overdetermined recovery setting. It can can be ameliorated by decreasing the number of considered measurements. In Fig. 4C the performance is shown for increasingly complex bias from rank-1 to rank-20. The necessary measurements required for improved recovery in the case of a worst-case correlation structure (e.g., maximally 2,500 possible measurements) are feasible to obtain up to a noise complexity of rank-9. In the best-case scenario (e.g., maximally 60,000 possible measurements) measurements are feasible to obtain up to at least rank-20. Notably, recovery is performed for matrix dimensions of 50 × 50 and thus the scaling behavior observed in Fig. 4B may improve performance depending on the size of the database considered. In Fig. 4D we evaluate blind recovery performance, where as opposed to k-sparse recovery with 2-sparse sensing matrices, entries are not sampled from a Gaussian distribution, but constructed *post hoc* from known or estimated correlations. For purposes of comparison with the k-sparse recovery based on 2-sparse sensing matrices, we force accurate estimation of correlations and corresponding standard deviations. No significant difference in performance between blind and 2-sparse recovery are observed for this setup, as shown in Figs. 4C–4D. Thus, recovery is feasible when the redundancies obtained in feature and sample space are estimated accurately and are sufficiently incoherent with the low-rank bias **X**. Discrepancies in perfect recovery between the bottom left of Figs. 4D and 4C are likely due to constraints in the construction of the measurement operator; only full rows and columns are considered for blind recovery in Fig. 4D, which for matrix dimensions of 50 × 50 create measurement increments of step size 50. Notably, these do not overlap exactly with the more fine grained scale of k-sparse recovery.

### Recovery robustness

**Figure 5:**
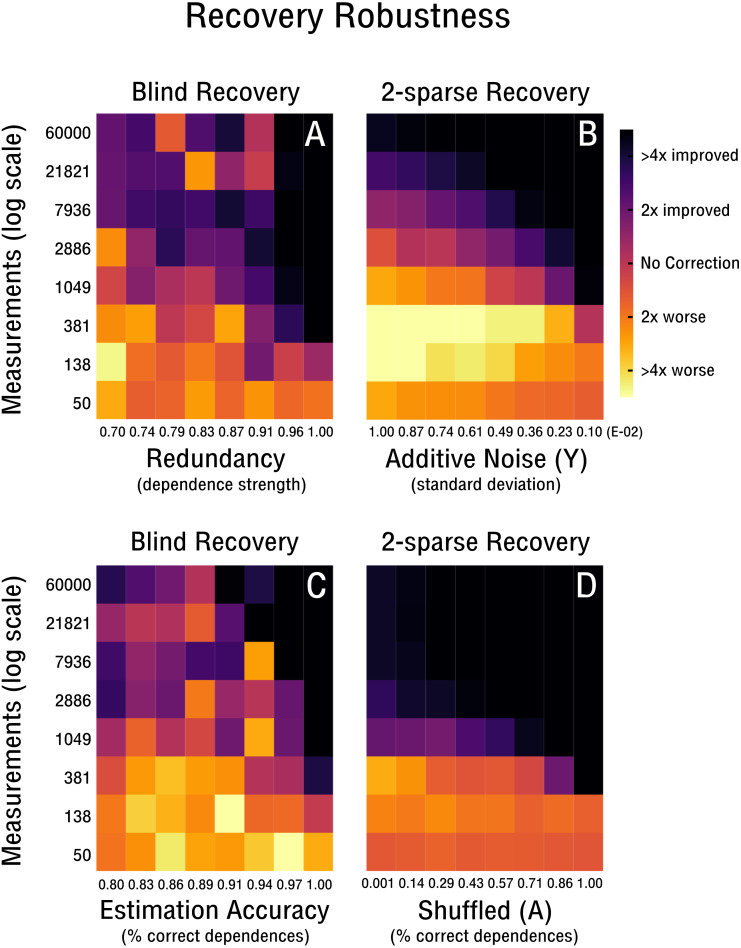
Robustness of blind and 2-sparse recovery. (A, B) As redundancy increases from weak (*ρ* = 0.7) to strong (*ρ* = 1.0) less measurements are required to blindly recover the low-rank bias (rank-2, 50 × 50). (C, D) As the accuracy of estimating signal redundancies from the confounded observations increases, the measurements required to blindly recover the low-rank bias (rank-2, 50 × 50) are reduced. The corresponding 2-sparse recovery is simulated for additive noise in **y** or shuffling in **A** to mimic the effect of varying redundancy and estimation accuracy for the non-blind case.

We continue our evaluation of blind recovery in Figs. 5A and 5D with a focus on recovery robustness. In particular, we observe that for the case of non-ideal redundancies, blind bias recovery is still feasible, as shown in Fig. 5A. Accordingly, as the redundant signal increases from weak redundancies (*ρ* = 0.7) to strong redundancies (*ρ* = 1.0) fewer measurements are necessary to blindly recover an unknown bias matrix (see Fig. 5A). Thus, blind recovery is somewhat robust to imperfect redundancies likely found in actual high-throughput databases. In Fig. 5C we observe that lower accuracy in the form of falsely estimated redundancies (e.g., wrong pairs of correlated features or samples) are recoverable up to a certain degree. In addition, we provide a comparison with k-sparse recovery for an identical setup, where redundancy and estimation accuracy are modeled as additive noise in Y (see Fig. 5B) and shuffled measurement operator A (see Fig. 5D). Both approaches perform well in the robustness evaluation, but it is difficult to align their scales for quantitative comparison.

### Benchmarking

In order to benchmark the developed blind recovery approach we mimic a standard research problem involving high-throughput data and compare to a widely used unsupervised normalization approach. The aim is to identify differentially expressed genes under different noise conditions at a given significance level (*p* = 0.05). For this purpose a high-throughput database is simulated as in ‘Simulation’ (see Data Availability). It contains 30 samples with 40 measured genes (features) each and two groups of replicates that are used to determine differential expression by a standard *t*-test. We force accurate estimation of correlations and corresponding standard deviations, as the small database size yields poor estimates that cause the recovery to be unstable for the limited number of available measurements (see Figs. 5A, 5C). The benchmark is performed across different noise conditions: random noise derived from $\mathcal{N}(0,1)$, systematic noise with rank-2 as outlined in ‘Simulation’ and no noise (see Table 1).

**Table 1:** Comparison of blind compressive normalization (BCN) with quantile normalization (QN) and no correction (NC) of the corrupted data. Data was corrupted with random, systematic and no noise. A *t*-test is performed between two groups of replicates (five each) for all genes (40 in total) and the resulting *p*-values are averaged. Plus (+) and minus (−) denote if the avg. *p*-value falls below the significance level of 0.05, where the expected avg. *p*-value for no noise and no correction is 2.04E–42.

	BCN	(avg. *p*-value)	QN	(avg. *p*-value)	NC	(avg. *p*-value)
Random Noise	−	3.42E–01	−	4.17E–01	−	3.89E–01
Systematic Noise	+	3.16E–02	−	1.66E–01	−	1.67E–01
No Noise	+	3.01E–03	+	3.64E–26	+	2.04E–42

In the case of random noise, both approaches perform similarly and are unable to reverse the effect of the corruption through normalization. Thus, no differentially expressed genes are detected at the given significance level (*p* = 0.05), which is expected. In the case of systematic noise, the blind compressive normalization (BCN) approach outperforms quantile normalization (QN) and is able to detect differential expression given the accurate estimation of correlations and corresponding standard deviations. In the case of no noise, no correction (NC) performs best, followed by the QN and BCN approach. Both approaches are able to detect differentially expressed genes for the case of no noise. Overall, this benchmark shows that the developed approach can outperform existing approaches on a standard research problem under idealized conditions.

## Discussion

A key aspect of the proposed algorithm for blind normalization of high-throughput databases is the sparsity assumption (see Introduction). By assuming that bias has a sparse structure, due to a limited number of confounding factors, the recovery problem becomes manageable and efficient optimization on manifolds can be used for recovery. The larger a high-throughput database is in size, the more effectively we can leverage the associated redundancies, since we can focus on correlations estimated with high-specificity and low-sensitivity. This is critical, as blind recovery requires a sufficient number of accurately estimated correlations. In addition, spike-in controls can provide further constraints on the bias to be recovered. These can be important sources of additional information to be leveraged by our approach, as integration through additional measurements via *entry* sensing is straight forward (see ‘K-sparse recovery’). But, it remains an open question how such absolute and relative constraints interact when solving the bias recovery problem (see Definition 1).

For the sparsity assumption to be of use for blind normalization, two further assumptions must be satisfied. Sufficient redundancies are needed in the form of correlations found in the high-throughput database at hand. This assumption is generally satisfied, since complex systems under study, such as the cell, generally display a number of strong correlations that are detectable despite the effect of confounding factors. In addition, high-throughput databases of a certain size are likely to contain redundancies in the form of similar biological samples that can be leveraged. Finally, blind normalization is only possible if the detected correlations are sufficiently incoherent with the low-dimensional bias model. The likelihood of such incoherence is maximized if correlated features and samples exhibit standard deviations similar to those drawn from a normal distribution, such as in the presented case of k-sparse recovery (see ‘K-sparse recovery’). In the setting of blind recovery, this assumption may only be satisfied for features and not for samples, as correlated samples have generally similar standard deviations. However, when evaluating recovery performance in simulation this does not appear to play a major role (see Figs. 4–5). A theoretical investigation of worst case performance and recovery guarantees is still outstanding, but recent work in the field of blind deconvolution and compressed sensing is in active pursuing this question (Stöger, Jung & Krahmer, 2016).

To scale the developed algorithm to current public high-throughput databases with features and samples on the order of hundred thousands respectively, the memory consumption of the underlying manifold optimization routines needs to be optimized to be efficient on the scale of gigabytes. However, the manifold optimization routines leveraged in our proof-of-concept implementation are not able to exploit the advantages that come with sparse measurement operators, e.g., a low-memory footprint. This is due to the use of conjugate gradient methods that rely on automatic differentiation (Maclaurin, Duvenaud & Adams, 2015) and require the use of memory inefficient dense matrices. The current implementation is thus only able to handle databases on the order of hundreds of features and samples respectively. Hence, an application outside of the scope of the conducted simulations is currently not feasible and should be addressed in future work. However, there appears to be no theoretical limitation that would preclude the development of a memory efficient implementation. This is important, since the proposed approach increases in effectiveness as database size grows and thereby allows the leveraging of more redundancies (see Fig. 4B).

An additional challenge exists when using fixed rank constraints in matrix recovery problems, as is the case for the employed manifold optimization routines. The fixed rank of the to be recovered low-rank matrix is generally not known *a priori*. Thus, optimization routines need to be run multiple times for different rank parameters in order to determine the optimal rank. This is an inefficient scheme when contrasted to recovery methods based on nuclear norm regularization (Mazumder, Hastie & Tibshirani, 2010). Furthermore, inappropriate choices of the rank parameter can result in ill-conditioned matrices for which manifold optimization routines may converge slowly. To address these challenges, a pursuit type scheme has been developed recently that can be understood as a warm start technique (Tan et al., 2014).

## Conclusion

Blind compressive normalization is a systematic approach to the blind normalization of unknown confounding factors in public high-throughput databases. The presented proof-of-concept shows that such an approach is possible under reasonable assumptions. Further work in this direction has the potential to address long standing challenges in high-throughput data integration that are becoming increasingly important.

## Additional Information and Declarations

### Data Availability

The following information was supplied regarding data availability: Source code is available at GitHub: https://github.com/a378ec99/bcn. Supplemental data is available at https://github.com/a378ec99/bcn/blob/master/resource/supplementary_data.zip.
